# *TNFRSF1B* Gene Variants in Clinicopathological Aspects and Prognosis of Patients with Cutaneous Melanoma

**DOI:** 10.3390/ijms25052868

**Published:** 2024-03-01

**Authors:** Bruna Fernandes Carvalho, Gabriela Vilas Bôas Gomez, Juliana Carron, Ligia Traldi Macedo, Gisele Melo Gonçalves, Vinicius de Lima Vazquez, Sergio Vicente Serrano, Gustavo Jacob Lourenço, Carmen Silvia Passos Lima

**Affiliations:** 1 Laboratory of Cancer Genetics, School of Medical Sciences, University of Campinas, Campinas 13083-888, SP, Brazil; brunafernandescarvalho@gmail.com (B.F.C.); gabivbg@gmail.com (G.V.B.G.); jcarron@unicamp.br (J.C.); ligiamed@gmail.com (L.T.M.); guslour@unicamp.br (G.J.L.); 2 Department of Anesthesiology, Oncology, and Radiology, School of Medical Sciences, University of Campinas, Campinas 13083-888, SP, Brazil; 3 Melanoma and Sarcoma Surgery Department, Barretos Cancer Hospital, Barretos 14784-400, SP, Brazil; gisagmg@hotmail.com (G.M.G.); viniciusvazquez@gmail.com (V.d.L.V.); 4 Department of Medical Oncology, Barretos Cancer Hospital, Barretos 14784-400, SP, Brazil; svserrano@hotmail.com

**Keywords:** cutaneous melanoma, *TNFRSF1B*, single nucleotide variant, clinicopathological aspects, survival

## Abstract

Regulatory T lymphocytes play a critical role in immune regulation and are involved in the aberrant cell elimination by facilitating tumor necrosis factor connection to the TNFR2 receptor, encoded by the *TNFRSF1B* polymorphic gene. We aimed to examine the effects of single nucleotide variants *TNFRSF1B* c.587T>G, c.*188A>G, c.*215C>T, and c.*922C>T on the clinicopathological characteristics and survival of cutaneous melanoma (CM) patients. Patients were genotyped using RT-PCR. *TNFRSF1B* levels were measured using qPCR. Luciferase reporter assay evaluated the interaction of miR-96 and miR-1271 with the 3′-UTR of *TNFRSF1B*. The c.587TT genotype was more common in patients younger than 54 years old than in older patients. Patients with c.*922CT or TT, c.587TG or GG + c.*922CT or TT genotypes, as well as those with the haplotype TATT, presented a higher risk of tumor progression and death due to the disease effects. Individuals with the c.*922TT genotype had a higher *TNFRSF1B* expression than those with the CC genotype. miR-1271 had less efficient binding with the 3′-UTR of the T allele when compared with the C allele of the SNV c.*922C>T. Our findings, for the first time, demonstrate that *TNFRSF1B* c.587T>G and c.*922C>T variants can serve as independent prognostic factors in CM patients.

## 1. Introduction

Cutaneous melanoma (CM) is a low-incidence tumor worldwide, but with variable lethal potential; patients with superficial skin lesions are generally cured of the disease, but those with metastatic lesions usually present short survival [1].

There are several significant risk factors considered in the development and progression of cutaneous melanoma (CM). Consequently, this disease is complex and multifactorial [2]. Environmental factors, such as skin exposure to ultraviolet (UV) radiation from sunlight and other sources [3], and genetic factors, including inherited mutations in cyclin-dependent kinase inhibitor 2A (*CDKN2A*), cyclin-dependent kinase 4 (*CDK4*), xeroderma pigmentosum (*XP*), and melanocortin 1 receptor (*MC1R*) [2,4], are frequently identified as factors related to CM. Moreover, CM is one of the most immunogenic types of solid tumors, eliciting an active antitumor response [5]. Thus, both acquired [6] and inherited [7] failures to recognize and combat abnormal melanocytes by the immune system may predispose individuals to the tumor or contribute to tumor progression. Different cells of the immune system are required for the destruction of abnormal cells [6,8,9], and lymphocytes are recognized as the primary cells in antitumor immunity [8,9]. Lymphocytes include NK cells (responsible for the destruction of abnormal cells and regulation of immune responses), B lymphocytes (engaged in antibody production and release), CD4 T lymphocytes (involved in the regulation of immune responses), and CD8 T lymphocytes (responsible for the direct destruction of abnormal cells). The latter two types of cells are also identified as regulatory T lymphocytes (Tregs) for immune response regulation and effector T lymphocytes (Teffs) [3,8,9].

Tregs play crucial roles in promoting immune tolerance, tumor development, and progression. These cells inhibit the activation and differentiation of CD4+ helper T cells and CD8+ cytotoxic T cells [10], block costimulatory signals from CD80 and CD86, regulate interleukin-2 (IL-2), secrete inhibitory cytokines, and kill Teffs [11,12]. In addition, Tregs modulate the destruction of abnormal cells by binding the tumor necrosis factor (TNF) to tumor necrosis factor receptor 2 (TNFR2) on their surfaces [11,13]. TNFR2 expression is upregulated in activated Tregs, and the binding of TNF to TNFR2 increases the proliferation, survival, and suppressive function of Tregs [14,15]. In B16F10 metastatic melanoma mice, TNF, through its binding to TNFR2, induced Treg proliferation, resulting in the escape of tumor cells from immune surveillance. Treg-cell depletion reduced the number of metastases in TNFR2-deficient animals [16]. Notably, basal levels of TNFR2 altered relapse-free survival in CM patients [17], and antibodies targeting TNFR2 on Tregs are seen as promising agents to promote immune-mediated control of tumors [18,19,20,21,22].

TNFR2 is encoded by the polymorphic gene *TNFRSF1B* [23]. Therefore, the ability to destroy abnormal cells varies among individuals, and can lead to distinct clinicopathological aspects and outcomes in CM patients.

The *TNFRSF1B* c.587T>G (rs1061622) single nucleotide variant (SNV) results in the replacement of thymine (T) by guanine (G) in the amino acid coding region, leading to the change in methionine by arginine at position 196 of the protein chain [24]. This variation does not appear to alter the TNF binding kinetics of TNFR2, but affects TNF-induced apoptosis in adenocarcinoma cervical cancer cells (HeLa cells) through impaired NF-kB signaling and target gene expression [25]. An exchange of adenine (A) by guanine (G) is determined by *TNFRSF1B* c.*188A>G (rs1061624) SNV [26], while *TNFRSF1B* c.*215C>T (rs3397) [26] and *TNFRSF1B* c.*922C>T (rs1061628) [27] SNVs result in the substitution of cytosine (C) by thymine (G). The roles of these SNVs in the encoded proteins remain unclear, but the last three mentioned SNVs, located in the 3′-untranslated region (3′-UTR) of the *TNFRSF1B*, can alter microRNA (miR) binding, influencing gene expression and CM progression [28]. The roles of these *TNFRSF1B* SNVs in the outcomes of patients with lung cancer [29], non-Hodgkin lymphoma [30], and esophageal carcinoma [31] remain controversial, and their impacts on the outcomes of patients with CM are unknown.

MiRs are small non-coding RNA molecules that predominantly bind to the 3′-UTR of messenger RNA (mRNA) of target genes, resulting in mRNA silencing or degradation and the inhibition of protein production [32,33]. Several miRs, such as miR-16, miR-204, miR-210, miR-221, and miR-222, play important roles in CM development and progression [34]. Additionally, miR-146a, miR-155, miR-125b, miR-100, let-7e, miR-125a, miR-146b, and miR-99b are associated with immune response modulation and therapy resistance in CM [35]. The *TNFRSF1B* is a target for miR binding; in silico analysis predicts direct targeting by miR-19a, miR-103a, miR-130a, and miR-17 in gastric cancer and lymphoma [36,37]. Furthermore, miR-148a binding at the *TNFRSF1B* modulated colitis-associated tumorigenesis in mice [38]. Understanding the functional role of different miRs in CM, identifying their direct targets, and elucidating their regulatory mechanisms are crucial, as abnormalities in the activity of these miRs contribute to tumor development and progression [39,40]. In the present study, we analyzed, for the first time, the associations of *TNFRSF1B* c.587T>G, c.*188A>G, c.*215C>T, and c.*922C>T SNVs with clinicopathological aspects and prognoses in CM patients. Additionally, we conducted functional studies to understand their biological consequences and associations.

## 2. Results

### 2.1. Study Population

Clinicopathological aspects of patients are presented in Table 1. The patients’ median age was 54 years old. Most patients were white-skinned, had more than 20 nevi, and their skin had been sun-exposed during their lifetime. Most melanomas were at stages I or II, had superficial spreading, had ≤1 or 2 mm of Breslow thickness, and were of Clark levels III or IV.

### 2.2. TNFRSF1B c.587T>G SNV at Age of Diagnosis

Patient samples were in the HWE at the loci of SNVs of the *TNFRSF1B* gene (c.587T>G: χ^2^ = 0.25, *p* = 0.61; c.*188A>G: χ^2^ = 1.07, *p* = 0.30; c.*215C>T: χ^2^ = 0.84, *p* = 0.35; c.*922C>T: χ^2^ = 1.86, *p* = 0.17). TGTC, TACC, and TATT haplotypes of the mentioned SNVs were considered for the study.

The frequencies of *TNFRSF1B* c.587T>G, c.*188A>G, c.*215C>T, and c.*922C>T genotypes in patients stratified by clinical aspects are presented in Table 2. The c.587TT genotype was more common in patients aged ≤54 years than in older patients (69.5 versus 57.0%, *p* = 0.007). Combined genotypes and haplotypes of the SNVs did not alter the clinical aspects of the patients.

No associations of c.587T>G, c.*188A>G, c.*215C>T, and c.*922C>T genotypes (Supplementary Table S1), combined genotypes, and haplotypes with pathological aspects of tumors were found in the study.

### 2.3. TNFRSF1B c.587T>G and c.*922C>T SNVs in Survival of Patients with CM

Clinical data from nine patients seen at the University of Campinas were inconsistent and they were excluded from the survival analysis. One hundred sixty-nine patients from the Pio XII Foundation were not included in the analysis of survival due to difficulties in updating the data imposed by the COVID-19 pandemic. Five-year (60 months) progression-free survival (PFS) and melanoma-specific survival (MSS) rates for patients with CM were 74.1% and 84.8%, respectively.

**Figure 1:**
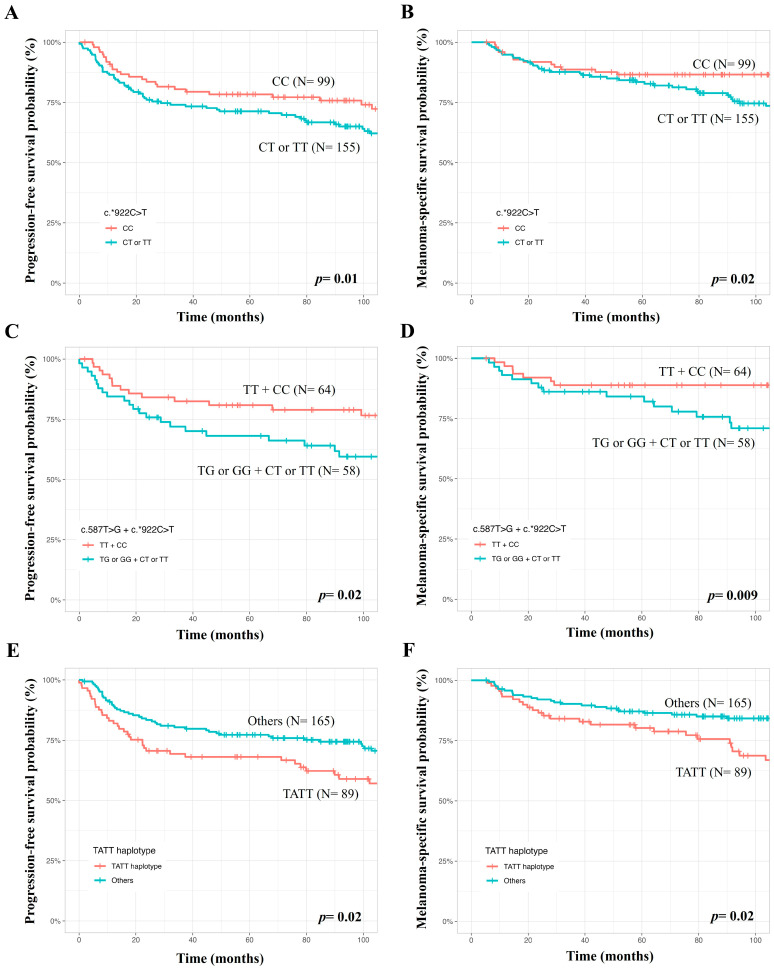
Analysis of genetic variants in the *TNFRSF1B* gene in survival of patients with cutaneous melanomas, where lower progression-free survival and melanoma-specific survival were seen in patients with the *TNFRSF1B* c.*922CT or TT genotypes (**A**,**B**), the *TNFRSF1B* c.587TG or GG + c.*922CT or TT genotypes (**C**,**D**), and TATT haplotype (**E**,**F**) compared to patients with the remaining genotypes and haplotypes.

At 60 months of follow up, PFS was lower in males (69.8 versus 78.4%, *p* = 0.03), patients with tumors at clinical stages III or IV (33.0 versus 82.7%, *p* < 0.0001), non-superficial spreading (59.6 versus 88.9%, *p* < 0.0001), T3 or T4 Breslow thickness values (49.6 versus 92.4%, *p* < 0.0001), and at Clark levels III to V (66.0 versus 93.4%, *p* < 0.0001) (Kaplan–Meier estimates). In Cox’s univariate analysis, males, patients with tumors at clinical stages III or IV, non-superficial spreading, T3 or T4 Breslow thickness values, and Clark levels III to V had more chances of presenting a progression of disease than others. Only a tendency for lower PFS was seen in patients with the TATT haplotype of *TNFRSF1B* SNVs. In Cox’s multivariate analysis, patients with tumors at stages III or IV, non-superficial spreading, T3 or T4 Breslow thickness values, c.*922CT or TT genotypes, c.587TG or GG + c.*922CT or TT genotypes, and TATT haplotypes of *TNFRSF1B* c.587T>G, c.*188A>G, c.*215C>T, and c.*922C>T SNVs had more chances of presenting disease progression than others (Table 3, Figure 1).

At 60 months of follow up, MSS was lower in patients aged >54 years (79.2 versus 90.3%, *p* = 0.007), males (77.4 versus 92.1%, *p* = 0.0003), patients with tumors at clinical stages III or IV (46.8 versus 92.0%, *p* < 0.0001), non-superficial spreading (73.5 versus 95.9%, *p* < 0.0001), T3 or T4 Breslow thickness values (68.9 versus 96.6%, *p* < 0.0001), and Clark levels III to V (78.8 versus 98.6%, *p* < 0.0001) (Kaplan–Meier estimates). In Cox’s univariate analysis, patients aged>54 years, males, patients with tumors at stages III or IV, non-superficial spreading, T3 or T4 Breslow thickness values, Clark levels III to V, and TATT haplotypes of *TNFRSF1B* c.587T>G, c.*188A>G, c.*215C>T, and c.*922C>T SNVs had more chances of evolving to death due to CM effects. In Cox’s multivariate analysis, patients aged>54 years, with tumors at clinical stages III or IV, non-superficial spreading, c.*922CT or TT genotypes, c.587TG or GG + c.*922CT or TT genotypes, and TATT haplotypes of *TNFRSF1B* SNVs had more chances of evolving to death due to CM (Table 3, Figure 1).

The remaining single/combined genotypes and haplotypes of the analyzed SNVs did not affect patients’ survival (Supplementary Table S2). 

### 2.4. TNFRSF1B c.*922C>T SNV in Gene Expression

Similar *TNFRSF1B* expressions were seen in individuals with different genotypes and alleles of c.587T>G SNV. 

**Figure 2:**
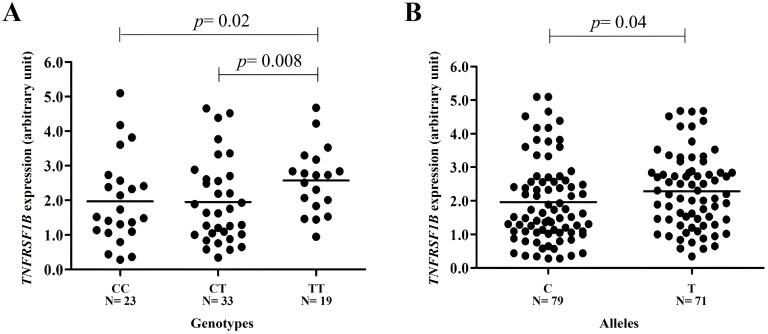
*TNFRSF1B* gene expression in peripheral blood leukocyte samples from individuals stratified by SNV c.*922C>T variant genotypes. The expression is higher in (**A**) individuals with the TT genotype when compared to individuals with CC (2.57 arbitrary units (AUs) ± 0.96 standard deviation (SD) versus 1.96 AUs ± 1.26 SD; *p* = 0.02) or CT (2.57 AUs ± 0.96 SD versus 1.94 AUs ± 1.21 SD; *p* = 0.008) genotypes and (**B**) in carriers of the variant T allele when compared to those with the ancestral C allele (2.28 AUs ± 1.11 SD versus 1.95 AUs ± 1.22 SD; *p* = 0.04). No difference in gene expression was observed in individuals with CC and CT genotypes (*p* > 0.05) (*t* test).

Individuals with the TT genotype of *TNFRSF1B* c.*922C>T presented a higher gene expression than those with the CC genotype (2.57 arbitrary units (AUs) ± 0.96 standard deviation (SD) versus 1.96 AUs ± 1.26 SD; *p* = 0.02) or CT (2.57 AUs ± 0.96 SD versus 1.94 AUs ± 1.21 SD; *p* = 0.008) genotypes. No difference in gene expression was observed in individuals with CC and CT genotypes (*p*>0.05). Gene expression was also higher in individuals with T alleles when compared to individuals with C alleles (2.28 AUs ± 1.11 SD versus 1.95 AUs ± 1.22 SD; *p* = 0.04) (Figure 2).

### 2.5. TNFRSF1B c.*922C>T SNV with miR-1271 in Luciferase Transcription

The relative luciferase activity did not show significant differences in SK-MEL-28 and A-375 cells co-transfected with the plasmid pMIR c.*922C and the inhibitory sequence of miR-96 when compared to the group containing the same plasmid co-transfected with the mimic sequence of miR-96 (SK-MEL-28: 100.0 versus 70.0%, *p* = 0.23; A-375: 100.0 versus 79.0%, *p* = 0.05). No differences were observed between the groups containing the plasmid pMIR_c.*922C co-transfected with the mimic sequence of miR-96 when compared to the group of pMIR_c.*922T plasmid co-transfected with the mimic sequence of miR-96 (SK-MEL-28: 70.0 versus 81.0%, *p* = 0.49; A-375: 79.0 versus 78.0%, *p* = 0.25) (Supplementary Figure S1).

**Figure 3:**
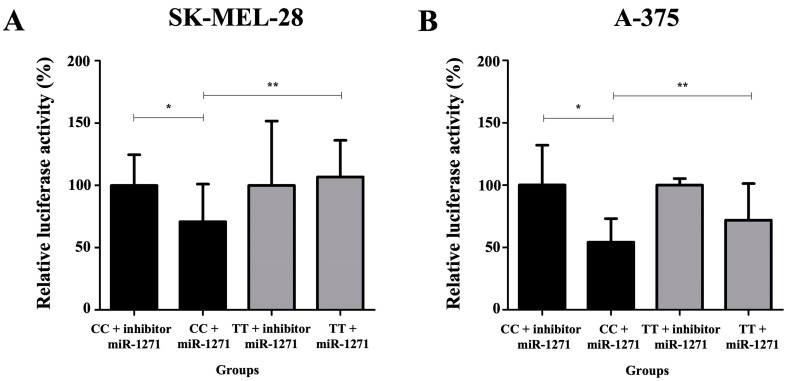
Quantification of relative luciferase activity in groups: pMIR c.*922C (CC genotype) co-transfected with microRNA inhibitor (miR)-1271 (considered 100% luciferase enzyme activity); pMIR c.*922C co-transfected with the mimic sequence of miR-1271; pMIR c.*922T (TT genotype) co-transfected with the inhibitor of miR-1271; and pMIR c.*922T co-transfected with the mimic sequence of miR-1271 in melanoma cell lines SK-MEL-28 (**A**) and A-375 (**B**). (*) The relative luciferase activity was higher when comparing the group containing the plasmid with the CC genotype co-transfected with the inhibitory sequence of miR-1271 and the group containing the same plasmid co-transfected with the mimic sequence of miR-1271 (SK-MEL-28: 100.0 versus 71.0%; *p* = 0.04; A-375: 100.0 versus 54.0%; *p* = 0.01). (**) The relative luciferase activity was lower when comparing the group containing the plasmid with the CC genotype co-transfected with the mimic sequence of miR-1271 and the group containing the plasmid TT co-transfected with the mimic sequence of miR-1271 (SK-MEL-28: 71.0 versus 107.0%, *p* = 0.02; A-375: 54.0 versus 72.0%, *p* = 0.01) (*t* test).

SK-MEL-28 and A-375 cells co-transfected with pMIR_c.*922C and miR-1271 inhibitor featured an increase in luciferase activity when compared with those co-transfected with pMIR_c.*922C and miR-1271 mimics (SK-MEL-28: 100.0 versus 71.0%; *p* = 0.04; A-375: 100.0 versus 54.0%; *p* = 0.01). SK-MEL-28 and A-375 cells co-transfected with pMIR_c.*922C and miR-1271 mimic sequences featured lower luciferase activity when compared with those co-transfected with pMIR c.*922T and miR-1271 mimic sequences (SK-MEL-28: 71.0 versus 107.0%, *p* = 0.02; A-375: 54.0 versus 72.0%, *p* = 0.01) (Figure 3). 

## 3. Discussion

In the current study, the roles of *TNFRSF1B* c.587T>G, c.*188A>G, c.*215C>T, and c.*922C>T SNVs in the clinicopathological aspects and survival of CM patients and expression of the *TNFRSF1B* gene were investigated. In addition, c.*922C>T SNV was analyzed by the luciferase reporter gene assay with the purpose of evaluating its interaction with miRs in disease.

It was initially observed that the clinical aspects of our patients and pathological aspects of the tumor were like those seen in patients analyzed in other parts of Brazil [41,42,43,44] and of the world [45,46,47,48,49]. Therefore, the sample enrolled in the current study was representative of CM, and consequently, it could be used for the assessment of factors associated with the clinicopathological aspects of CM and prognosis of patients with CM.

Secondly, it was found that the TT genotype of *TNFRSF1B* c.587T>G SNV was more common in younger patients (patients aged ≤54 years compared to older patients), a common aspect of tumors with unequivocal genetic components, such as those of hereditary syndromes [50,51] and familial tumors [52,53], which are, respectively, determined by high and low penetrance mutations. 

To the best of our knowledge, there are no previous studies focusing on the role of *TNFRSF1B* c.587T>G SNV in the clinicopathological aspects of cancer patients, and therefore, it was not possible to compare our data with others in the literature. Similar *TNFRSF1B* expression was seen in the peripheral blood samples of individuals with different genotypes and alleles of c.587T>G SNV enrolled in the current study. Nevertheless, the observation of an excess of the *TNFRSF1B* c.587 TT genotype in patients aged ≤ 54 years was not exactly a surprise, since the SNPs3D algorithm predicted a possible alteration in TNFR2 stability by the SNV. It was previously described that Tregs inhibit Teffs, and Tregs in normal tissue can be predisposed to tumor occurrence, and, in the tumor microenvironment, can induce tumor progression and spread [11]. TNF was produced by 83% of primary melanomas and 57% of metastatic melanomas [54]. In addition, TNF, through its binding to TNFR2, induced Tregs proliferation in B16F10 mice with metastatic melanomas, resulting in the escape of tumor cells from immune surveillance, and Treg cell depletion in TNFR2-deficient animals reduced the number of metastases of CM [9]. With these findings and descriptions, we hypothesized that the c.587TT genotype of *TNFRSF1B* SNV could guarantee the stability of TNFR2, favoring TNF binding, and inducing Treg proliferation and Teffs inhibition in the skin, favoring the shorter survival of abnormal melanocytes.

Third, shorter PFS and MSS were observed in patients with c.*922CT or TT genotypes, c.587TG or GG + c.*922CT or TT combined genotypes, and TATT haplotypes of *TNFRSF1B* c.587T>G, c.*188A>G, c.*215C>T, and c.*922C>T SNVs. In the Cox multivariate analysis, patients with the respective genotypes and haplotypes presented 2.00, 2.48, and 1.75 more chances of presenting a progression of disease than others, and 2.09, 3.68, and 1.98 more chances of evolving to death due to CM, respectively. To our knowledge, there are no previous studies focusing on the association of *TNFRSF1B* c.*922C>T SNV with outcomes of cancer patients. The *TNFRSF1B* c.587GG genotype was associated with a better OS of patients with lung cancer [29]. The *TNFRSF1B* c.587T>G genotype did not alter the responses to 5-fluorouracil and cisplatin and survival of patients with esophageal carcinomas [31], but its role in the CM outcome is still unknown. Thus, our data indicate that the c.*922C>T and c.587GG SNVs can alter the survival of CM patients. Other immune-related variants enrolled in the regulation of T-lymphocyte activity, the PD1.1 and PD1.5 SNVs of the *PDCD1* gene, also altered the recurrence-free survival of CM and provided support to the findings of the current study [7].

The association of *TNFRSF1B* c.*922CT or TT genotypes with shorter PFS and MSS was expected in patients in the current study. In fact, a high *TNFRSF1B* expression was found in individuals with the TT genotype of the c.*922C>T SNV, which could have resulted from the less efficient binding of miR-1271 with the *TNFRSF1B* 3′-UTR region encoded by the T allele than by the C allele of c.*922C>T SNV. This finding may have guaranteed the presence of TNFR2 in Tregs of a tumor microenvironment. Furthermore, the binding of TNF to TNFR2 may have induced Tregs proliferation, Teffs inhibition, and the survival of abnormal melanocytes, resulting in the progression of CM. High basal levels of TNFR2 were previously associated with lower relapse-free survival in CM patients [17], and miR-1271 was previously associated with the inhibition of cell growth in lung cancer through mTor suppression [55], colorectal cancer by downregulating methaderin/Wnt signaling [56], and prostate cancer by the inhibition of the MAPK pathway [57]. However, the association of *TNFRSF1B* expression/TNFR2 levels, miRNA-1271, and *TNFRSF1B* c.*922C>T SNV with melanoma cell proliferation should be investigated by additional functional studies.

The role of c.587TG or GG genotypes of *TNFRSF1B* c.587T>G SNV in association with c.587TG or GG + c.*922CT or TT genotypes with shorter PFS and MSS is not easily explained. Similar *TNFRSF1B* expression was observed in individuals with *TNFRSF1B* c.587TT and GG genotypes in the current study. Till et al. (2005) [25] showed that the G allele determined a significantly lower capability to induce TNFR2-mediated NF-kB activation, and the pretriggering of TNFR2 with a receptor-specific mutein led to an enhancement of the TNFR1-induced apoptosis of transformed Hela cells carrying the GG genotype. However, it has recently been reported that TNFR2 is expressed by at least 25 types of tumors, including CM [58]. In colorectal cancer tissue, the expression of TNFR2 was correlated with Ki-67 expression [59] and a resistance to adriamycin in breast cancer [60], but the functional consequences of TNFR2 expression and of *TNFRSF1B* c.587T>G SNV on CM cells remain to be elucidated. The high expression of TNFR2 by tumor tissues has been viewed with enthusiasm because it is a premise to explore the possibility of tumor treatment with TNFR2-targeting agents [59]. Nevertheless, single c.587T>G SNV did not alter patients’ survival and did not increase the risk for disease relapse, disease progression, or death when associated with c.*922C>T SNV, and therefore, it is possible that the association of the combined genotype with the poor prognosis of CM patients is attributable only to the effect of c.*922C>T SNV.

We are aware that the results of the association of genotypes with the clinicopathological aspects and survival of CM patients have been obtained from a relatively small number of patients; *TNFRSF1B* c.587T>G genotypes were analyzed only in patients aged ≤54 years or >54 years; *TNFRSF1B* expression was measured only in the peripheral blood samples of individuals with distinct genotypes of c.587T>G and c.*922C>T SNVs; and the relation of genotypes of *TNFRSF1B* SNVs with TNFR2 quantity and quality in Tregs and CM cells, as well as their mechanisms of actions in CM, needs to be investigated further. 

Thus, the results obtained in the current study should be validated in further larger studies and complemented by mouse models of CM and functional studies. We also believe that a future study of patients with CM with different genotypes of the *TNFRSF1B* gene treated with target therapy (BRAF and MEK inhibitors) and immunotherapy (anti-PD1/PDL1 and anti-CTLA4) should be conducted to verify whether the new agents can overturn the unfavorable prognosis of the identified genotypes.

## 4. Materials and Methods

### 4.1. Study Population 

A total of 433 patients with CM were evaluated, with 264 patients diagnosed and treated at the Clinical Oncology Service of the General Hospital of the University of Campinas and 169 patients at the Cancer Hospital of Barretos of the Pio XII Foundation, during the period from November 2018 to July 2000. Patients with the amelonocytic or acral subtype were excluded from the study due to their distinct histological, phenotypic, genetic, and biological aspects when compared to other types of CM. The study received approval from the research ethics committees of the University of Campinas (process number 3.498.678) and the Pio XII Foundation (process number 3.646.661), and all procedures were conducted in accordance with the Helsinki Declaration.

### 4.2. Clinical Aspects, Tumor Aspects, and Treatment

Clinical characteristics of patients with CM were obtained through specific questionnaires. Skin color and the number of nevi were classified according to the International Agency for Research on Cancer (IARC) protocol [61], and skin phototype was identified using previously defined criteria [62]. Regarding sun exposure, patients were stratified as sun-exposed when exposed to more than two hours of sunlight per day for over ten years and non-sun-exposed when exposed to fewer hours and/or a shorter time interval in years [63]. Patients who reported pain and erythema, with or without blister formations, for more than 24 h in at least one event in their lifetime were classified as having experienced sunburn [64].

Tumor characteristics were obtained from the medical records of patients with CM. Tumors were classified based on previously defined criteria for location [65]. The diagnosis of CM was established through an anatomopathological examination using conventional criteria, with the histological type, Breslow index, and Clark level derived from the patient’s anatomopathological examination report. Tumor stage was determined following the criteria of the American Joint Committee on Cancer [66].

Patients were treated based on the protocol of the institutions [67]. In summary, patients initially underwent a local excision of the tumor as a diagnostic procedure, with a subsequent margin expansion when necessary. Sentinel lymph node evaluations were performed on patients with tumors having a Breslow index of more than 1 mm, and a lymphadenectomy was performed on those with a histological tumor infiltration or clinically positive lymph nodes. Patients with a single metastasis or operable recurrence underwent a surgical removal of the tumor, while those with multiple metastases and inoperable recurrences received chemotherapy with dacarbazine. Patients with unresectable brain and bone metastases underwent palliative treatment with radiotherapy. Patient treatment and follow-up data were obtained from the medical records.

### 4.3. Selection of SNVs and Associated miRNAs

A search in the database of the National Center for Biotechnology Information (www.ncbi.nlm.nih.gov/projects/SNP), accessed on 8 May 2021, was conducted, resulting in the identification of 38 SNVs in the *TNFRSF1B* gene for the present study. Subsequently, 9 SNVs previously associated with cancer were selected (Supplementary Table S3), and six SNVs with sample sizes compatible with the number of samples available at our laboratory (N = 550) remained in the study. One SNV located in the amino acid coding region (c.587T>G, rs1061622) and three SNVs in the 3′-UTR region (c.*188A>G, rs1061624; c.*215C>T, rs3397; and c.*922C>T, rs1061628) of the *TNFRSF1B* were chosen for the study (N = 4) due to potential structural alterations in the receptor and possible changes in miRNA binding sites, influencing gene expression, respectively. Frequencies of selected SNV genotypes, both isolated and in combination, were analyzed in patients and controls, as well as in patients stratified by clinicopathological features. The steps of SNV selection are illustrated in Supplementary Figure S2.

For the *TNFRSF1B* c.587T>G (rs1061622) SNV, in silico tests using PolyPhen-2 [68], SIFT, and SNPs3D [69] computer programs were conducted to verify a possible structural change in the TNFR2 receptor. It was observed that the prediction of a structural alteration in the receptor was called “benign” or “tolerated” by the PolyPhen-2 and SIFT algorithms. However, the stability of TNFR2 could be altered by the variant SNV allele, according to the analysis of SNPs3D (support vector machine profile −0.35). A score less than 0 meant the SNV could be predicted as deleterious [70].

Considering *TNFRSF1B* c.*188G>A, c.*215T>C, and c.*922C>T SNVs, in silico analyses using the SNPinfo [71], MicroSNiPer [72], and MirSNPscore [73] computer programs were performed to predict and select miRs associated with the possible change in the binding site on the 3′-UTR of the gene determined by SNVs. It was observed that c*188A>G SNV could change the binding site of eight miRs, c.*215T>C of 12 miRNAs, and c.*922C>T of 14 miRNAs (Supplementary Table S4). For further analysis, miRs associated with carcinogenesis processes and with a higher binding efficiency to the 3′-UTR regions of *TNFRSF1B* (seed-match sites: 8mer, 7mer-m8, 7mer-A1, 6mer, or 6mer offset) were selected [74] (Supplementary Table S4).

Three out of eight miRs associated with SNV c.*188A>G were related to tumor development and progression, but only miR-639 showed a higher binding efficiency (7mer-m8 binding), and it was seen as a potential candidate for functional analysis. Seven out of 12 miRNAs associated with c.*215C>T SNV were described in tumors, and miR-329-3p (7mer-m8 binding) and miR-362-3p (7mer-m8 binding) were considered the most interesting candidates for the study. Nine out of 14 miRs associated with c.*922C>T SNV were described in tumors; miR-96 (6mer binding) and miR-1271 (6mer binding) showed a higher association with tumors, and that was why they were selected as candidates for functional assays (Supplementary Figure S3).

### 4.4. Complementary Functional Study of Modified SK-MEL-28 and A-375 Cell Lines

SK-MEL-28 and A-375 melanoma cell lines, obtained from the Rio de Janeiro cell bank (Rio de Janeiro, Brazil) with Short Tandem Repeat (STR) analysis, were selected for the study. These cells (both *BRAF* mutated) were chosen due to their well-known molecular characterization, ease of cultivation, and genetic transformation. *TNFRSF1B* c.*922C>T was considered the SNV of greatest interest in the study due to its association with patients’ survival. To obtain melanoma cells with the same characteristics and genetic profiles, but expressing the ancestral or variant genotypes of c.*922C>T SNV for functional studies, a genetic transformation was performed. Modified SK-MEL-28 and A-375 cells with c.*922CC or TT genotypes were used in luciferase assay.

SK-MEL-28 and A-375 cells were cultured in Dulbecco’s Modified Eagle’s Medium (DMEM) (Gibco, Waltham, MA, USA) with 10% fetal bovine serum (FBS) and 1% penicillin–streptomycin (100 U/mL) (Gibco, Waltham, MA, USA), and were stored in a humidified atmosphere with 5% CO_2_ at 37 °C.

### 4.5. Determination of SNV Genotypes

Genotyping was performed in DNA extracted from peripheral blood leukocytes of the patients and controls. Genotypes of each SNV were obtained by real-time polymerase chain reaction (RT-PCR), using TaqMan^®^ SNP Genotyping Assay (Applied Biosystems^®^, Waltham, MA, USA; *TNFRSF1B* c.587T>G, C_8861232_20; *TNFRSF1B* c.*188A>G, C_8861229_10; *TNFRSF1B* c.*215C>T, C_8861228_20; *TNFRSF1B* c.*922C>T, C_3143033_10), following the manufacturer’s instructions. Positive and negative controls were used in all reactions. As previously reported [75], 15% of the samples (randomly chosen) were genotyped again in an independent experiment to guarantee the genotyping quality, and a 100% concordance rate was obtained.

### 4.6. TNFRSF1B Expression by Quantitative PCR (qPCR)

*TNFRSF1B* c.587T>G and c.*922C>T SNVs were selected for gene expression analyses due to their associations with patients’ clinicopathological aspects and survival. Total RNA was obtained from peripheral blood samples of 75 individuals with TT (N = 52), TG (N = 22), and GG (N = 1) genotypes of *TNFRSF1B* c.587T>G SNV, and from 75 individuals with CC (N = 23), TC (N = 33), and TT (N = 19) genotypes of c.*922C>T SNV. Subsequently, cDNA was synthesized using Superscript III RT reagents (Invitrogen, Waltham, MA, USA).

Gene expression was determined by qPCR using the SYBR Green PCR Master Mix reagent (Invitrogen, Waltham, MA, USA) and specific primers for the *TNFRSF1B* gene (forward: 5′-GG- TCATGAGTCCTTCCACGATAC-3′, and reverse: 5′-GTGTGTTGGGATCGTGTGGA-3′). The experiment was carried out with samples in triplicate and with a negative control. Gene expression was normalized considering the expression of the actin beta gene (forward: 5′-AAGAGATGGCCACGGCTGCT-3′, and reverse 5′-TCGCTCCAACCGACTGCTGT-3′) and calculated by applying the arithmetic formula 2^−ΔΔCT^ [76]. The values of gene expression were presented in AUs.

### 4.7. Plasmid Construction

As c.*922C>T was considered for functional studies, miR-96 and miR-1271 were selected for further analysis using luciferase assays.

Firstly, the 3′-UTR of the c.*922C (ancestral allele) and c.*922T (variant allele) mRNA (507 bps) of individuals with known genotypes (c.*922CC and c.*922TT, respectively) were amplified by PCR. Specific reagents and primers with restriction sites for *Sac*I and *Mlu*I enzymes were used: forward primer: 5′-GAGCTCGCACCTATAGTCC-CAG-3′ (*Sac*I enzyme restriction site is underlined) and reverse: 5′-ACGCGTGA-GGTAGGAG-TAGAGAG-3′ (*Mlu*I enzyme restriction site is underlined). The chosen restriction sites allowed the correct insertion of fragments to the pMIR-REPORT miRNA Expression Reporter Vector (Ambion, Waltham, MA, USA) using standard protocols. After the procedures, the plasmids pMIR_c.*922C and pMIR_c.*922T were obtained.

### 4.8. Dual-Luciferase Reporter Assay

SK-MEL-28 and A-375 cells were transiently transfected with plasmids pMIR_c.*922C (ancestral allele), pMIR_c.*922T (variant allele), *Renilla* Luciferase Control Reporter (pRL) (Promega, Madison, WI, USA) (normalizing control), and synthetic sequences of miR-96 and miR-1271 mimics, and inhibitor mimics (Ambion, Waltham, MA, USA), using Lipofectamine 3000 (Invitrogen, Waltham, MA, USA), according to the manufacturer’s instructions. Cells were harvested 48 h after transfection and the luciferase activity was measured by the Dual-Luciferase Reporter Assay System kit (Promega, Madison, WI, USA), according to the manufacturer’s instructions. Luciferase activity was normalized to the pRL vector activity. The tests were performed in triplicate and as three independent experiments.

### 4.9. Statistical Analysis

The Hardy–Weinberg equilibrium (HWE) was calculated using the chi-square test (χ^2^), where it was possible to verify whether there was a preferential distribution of any of the genotypes in the patient group. Haploview 4.2 software (www.broad.mit.edu/mpg/haploview), accessed on 17 March 2022, was used to select markers included in the haplotype analysis. Differences between groups of patients were analyzed using Fisher’s or chi-square tests. Differences in gene expression were analyzed by the *t* test. Furthermore, to adjust the values related to multiple comparisons of SNVs (clinical and biological aspects of the tumor), the Bonferroni method was used.

PFS was calculated from the date of surgery until the date of first recurrence, progression of disease, death from any cause, or last follow up. MSS was calculated from the date of diagnosis until the date of death from the effects of the disease or last follow up. PFS and MSS times were calculated using Kaplan–Meier probabilities, and differences between curves were assessed by the log-rank test. The prognostic impacts of clinicopathological aspects, genotypes, and haplotypes of SNVs were evaluated by the Cox univariate regression test, and subsequently, all variables with *p* < 0.20 were included in the Cox multivariate analysis. Survival analysis was performed in August 2023.

Significant results were considered when values of *p* were ≤0.05. All analyses were performed using the SPSS 21.0 statistical program.

## 5. Conclusions

In summary, the data presented here for the first time show that *TNFRSF1B* c.587T>G and c.*922C>T SNV can impact the clinicopathological features of CM and can act as independent prognostic factors in CM patients, and can be used in the future to select CM patients homogeneously treated for differentiated approaches.

## Figures and Tables

**Table 1:** Distribution of 433 cutaneous melanoma patients stratified by clinicopathological characteristics.

Variables	Patients N (%) or Median (Range)
Median age (years)	
≤54	226 (52.2)
>54	207 (47.8)
Gender	
Male	201 (46.4)
Female	232 (53.6)
Skin color	
White	403 (93.1)
Non-white	30 (6.9)
Nevi *	
<20	167 (38.6)
≥20	260 (60.1)
Not obtained	6 (1.3)
Sun exposure *	
Yes	349 (80.6)
No	73 (16.9)
Not obtained	11 (2.5)
Tumor location *	
Head or neck	77 (17.8)
Upper limb	64 (14.8)
Lower limb	78 (18.0)
Trunk	194 (44.8)
Not obtained	20 (4.6)
Clinical stage *	
0	36 (8.3)
I	193 (44.6)
II	131 (30.3)
III	53 (12.2)
IV	14 (3.2)
Not obtained	6 (1.4)
Histological type *	
Superficial spreading	254 (58.7)
Nodular	103 (23.8)
Malignant lentigo	34 (7.9)
No specification	42 (9.7)
Breslow thickness *	
T1	188 (43.4)
T2	71 (16.4)
T3	72 (16.6)
T4	85 (19.6)
Not obtained	17 (4.0)
Clark level *	
I	38 (8.8)
II	71 (16.4)
III	130 (30.0)
IV	161 (37.2)
V	19 (4.4)
Not obtained	14 (3.2)
Follow-up time **	96 (5–251)
Patient’s final status **	
Alive without melanoma	162
Alive with melanoma	15
Died from melanoma	55
Died from other causes	23

N: number of individuals; %: percentage. Breslow thickness: T1: ≤1.00 mm, T2: 1.00–2.00 mm, T3: 2.01–4.00 mm, and T4: >4.00 mm. *, **: the number of individuals differed from the initial one because it was not possible to obtain pertinent information in all cases. Cases of in situ melanoma were included in the no-specification group.

**Table 2:** Frequencies of genotypes of the *TNFRSF1B* single nucleotide variants in 433 cutaneous melanoma patients stratified by clinical aspects.

Genotypes	N	Median Age (Years)		Gender		Skin Color		Number of Nevi *		Sun Exposure *	
		≤54 N (%)	>54 N (%)	Male N (%)	Female N (%)	White N (%)	Non-White N (%)	<20 N (%)	≥20 N (%)	Yes N (%)	No N (%)
c.587T>G											
TT	275	157 (69.5)	118 (57.0)	127 (63.2)	148 (63.8)	252 (62.5)	23 (76.7)	102 (61.1)	169 (65.0)	222 (63.6)	48 (65.8)
TG or GG	158	69 (30.5)	89 (43.0)	74 (36.8)	84 (36.2)	151 (37.5)	7 (23.3)	65 (38.9)	91 (35.0)	127 (36.4)	25 (34.2)
*p*-value		**0.007**		0.89		0.16		0.41		0.72	
TT or TG	413	214 (94.7)	199 (96.1)	188 (93.5)	225 (97.0)	385 (95.5)	28 (93.3)	160 (95.8)	247 (95.0)	334 (95.7)	69 (94.5)
GG	20	12 (5.3)	8 (3.9)	13 (6.5)	7 (3.0)	18 (4.5)	2 (6.6)	7 (4.2)	13 (5.0)	15 (4.3)	4 (5.5)
*p*-value		0.50		0.10		0.64		0.81		0.75	
c.*188G>A *											
GG	142	75 (52.8)	67 (47.2)	75 (37.5)	67 (28.9)	130 (32.3)	12 (40.0)	53 (31.7)	87 (33.6)	118 (33.9)	20 (27.4)
GA or AA	290	151 (52.1)	139 (47.9)	125 (62.5)	165 (71.1)	272 (67.7)	18 (60.0)	114 (68.3)	172 (66.4)	230 (66.1)	53 (72.6)
*p*-value		0.88		0.05		0.38		0.69		0.28	
GG or GA	344	182 (52.9)	162 (47.1)	159 (79.5)	185 (79.7)	322 (80.1)	22 (73.3)	132 (79.0)	208 (80.3)	278 (79.9)	59 (80.8)
AA	88	44 (50.0)	44 (50.0)	41 (20.5)	47 (20.3)	80 (19.9)	8 (26.7)	35 (21.0)	51 (19.7)	70 (20.1)	14 (19.2)
*p*-value		0.62		0.95		0.35		0.75		0.85	
c.*215T>C											
TT	178	88 (38.9)	90 (43.7)	81 (40.3)	97 (41.8)	169 (41.9)	9 (30.0)	70 (41.9)	106 (40.8)	141 (40.4)	31 (42.5)
TC or CC	255	138 (61.1)	117 (56.5)	120 (59.7)	135 (58.2)	234 (58.1)	21 (70.0)	97 (58.1)	154 (59.2)	208 (59.6)	42 (57.5)
*p*-value		0.33		0.75		0.20		0.81		0.74	
TT or TC	384	195 (86.3)	189 (91.3)	176 (87.6)	208 (89.7)	358 (88.8)	26 (86.7)	149 (89.2)	230 (88.5)	309 (88.5)	65 (89.0)
CC	49	31 (13.7)	18 (8.7)	25 (12.4)	24 (10.3)	45 (11.2)	4 (13.3)	18 (10.8)	30 (11.5)	40 (11.5)	8 (11.0)
*p*-value		0.09		0.49		0.71		0.80		0.90	
c.*922C>T *											
CC	164	86 (38.2)	78 (37.7)	76 (37.8)	88 (38.1)	153 (38.1)	11 (36.7)	58 (34.7)	102 (39.4)	135 (38.7)	27 (37.0)
CT or TT	268	139 (61.8)	129 (62.3)	125 (62.2)	143 (61.9)	249 (61.9)	19 (63.3)	109 (65.3)	157 (60.6)	214 (61.3)	46 (63.0)
*p*-value		0.90		0.95		0.87		0.33		0.78	
CC or CT	379	199 (88.4)	180 (87.0)	179 (89.1)	200 (86.6)	353 (87.8)	26 (86.7)	148 (88.6)	225 (86.9)	307 (88.0)	65 (89.0)
TT	53	26 (11.6)	27 (13.0)	22 (10.9)	31 (13.4)	49 (12.2)	4 (13.3)	19 (11.4)	34 (13.1)	42 (12.0)	8 (11.0)
*p*-value		0.63		0.43		0.77		0.59		1.00	

N: number of patients; %: percentage. *: the number of patients differed from the initial one because it was not possible to obtain pertinent information in some cases. Nomenclatures of c.*188G>A (also known as c.*188A>G) and c.*215T>C (also known as c.*215C>T) single nucleotide variants change due to higher frequencies of G and T alleles seen in our population, respectively. * Significant *p*-value after the Bonferroni correction (*p* ≤ 0.01) is presented in bold.

**Table 3:** Significant clinicopathological aspects, genotypes, and haplotypes of the *TNFRSF1B* single nucleotide variants in survival of 255 cutaneous melanoma patients.

Variable	Univariate Cox Regression						Multivariate Cox Regression			
	N Total/ N Event	PFS HR (95% CI)	*p*-Value	N Total/ N Event	MSS HR (95% CI)	*p*-Value	PFS Adjusted HR (95% CI)	*p*-Value	MSS Adjusted HR (95% CI)	*p*-Value
Median age (years)										
≤54	125/38	Reference	0.15	125/22	Reference	0.009	Reference	0.74	Reference	**0.03**
>54	130/49	1.36 (0.89–2.08)		130/39	2.01 (1.18–3.39)		1.08 (0.65–1.81)		2.02 (1.06–3.84)	
Gender										
Male	129/52	1.58 (1.03–2.43)	0.03	129/43	2.61 (1.50–4.53)	0.0006	1.05 (0.56–1.58)	0.82	1.72 (0.91–3.27)	0.09
Female	126/35	Reference		126/18	Reference		Reference		Reference	
TNM stage										
0-II	206/53	Reference	<0.0001	206/36	Reference	<0.0001	Reference	**0.0001**	Reference	**<0.0001**
III or IV	43/31	4.65 (2.96–7.30)		43/25	5.47 (3.27–9.15)		2.99 (1.68–5.31)		4.71 (2.42–9.15)	
Type of tumor spread										
Superficial spreading	128/20	Reference	<0.0001	128/11	Reference	<0.0001	Reference	**0.01**	Reference	**0.0004**
Others	93/48	4.22 (2.50–7.13)		93/37	5.77 (2.94–11.33)		2.17 (1.19–3.96)		3.70 (1.77–7.72)	
Breslow thickness										
T1 or T2	147/23	Reference	<0.0001	147/16	Reference	<0.0001	Reference		Reference	
T3 or T4	94/54	5.19 (3.18–8.48)		94/39	4.65 (2.59–8.33)		2.19 (1.08–4.42)	**0.02**	1.15 (0.51–2.58)	0.73
Clark level										
I or II	77/8	Reference	<0.0001	77/4	Reference	0.0001	Reference		Reference	
III-V	165/72	5.33 (2.56–11.08)		165/53	7.35 (2.65–20.33)		2.06 (0.85–5.00)	0.10	3.65 (1.00–13.29)	0.05
c.*922C>T										
CC	99/29	Reference	0.16	99/19	Reference	0.13	Reference	**0.01**	Reference	**0.02**
CT or TT	155/58	1.36 (0.87–2.13)		155/42	1.51 (0.87–2.59)		2.00 (1.16–3.44)		2.09 (1.09–3.99)	
CC or CT	226/77	Reference	0.99	226/54	Reference	0.83	NA		NA	
TT	28/10	1.00 (0.51–1.92)		28/7	1.08 (0.49–2.38)					
c.587T>G + c.*922C>T										
TT + CC	64/18	Reference	0.13	64/12	Reference	0.16	Reference	**0.02**	Reference	**0.009**
TG or GG + CT or TT	58/23	1.59 (0.85–2.95)		58/16	1.71 (0.80–3.62)		2.48 (1.10–5.61)		3.68 (1.37–9.92)	
TT or TG + CC or CT	219/74	Reference	0.68	219/52	Reference	0.25				
GG + TT	4/2	1.34 (0.32–5.48)		4/2	2.26 (0.54–9.32)		NA		NA	
c.*215T>C + c.*922C>T										
TT + CC	28/6	Reference	0.20	28/2	Reference	0.09	Reference	0.17	Reference	0.17
TC or CC + CT or TT	81/30	1.73 (0.72–4.17)		81/21	3.50 (0.81–14.99)		2.16 (0.71–6.56)		3.11 (0.59–16.35)	
TT or TC + CC or CT	198/68	Reference	NC	198/47	Reference	NC	NA		NA	
CC + TT	1/0	NC		1/0	NC					
Haplotype										
TATT	89/36	1.49 (0.43–1.02)	0.06	89/28	1.77 (1.08–2.97)	0.02	1.75 (1.09–2.99)	**0.02**	1.98 (1.08–3.50)	**0.02**
Others	165/51	Reference		165/33	Reference		Reference		Reference	

N: number of patients; PFS: progression-free survival; MSS: melanoma-specific survival; HRs: relative risks of events; CI: confidence interval; Breslow thickness: T1 or T2: ≤2.00 mm and T3 or T4:>2.00 mm; NA: characteristics not included in multivariate analysis; NC: not calculated. Nomenclatures of c.*188G>A (also known as c.*188A>G) and c.*215T>C (also known as c.*215C>T) single nucleotide variants (SNVs) change due to the higher frequency of G and T alleles in our population, respectively. Haplotypes represent alleles of *TNFRSF1B* c.587T>G, c.*188G>A, c.*215T>C, and c.*922C>T SNVs. In type of tumor spread, nodular and malignant lentigines were classified as others. Significant *p*-values are presented in bold in the multivariate analysis.

## Data Availability

The data that support the findings of this study are available from the corresponding author upon reasonable request.

## Supplementary Materials

The following supporting information can be downloaded at: https://www.mdpi.com/article/10.3390/ijms25052868/s1. References [77,78,79,80,81,82,83,84,85,86,87,88,89,90,91,92,93,94,95,96,97,98,99,100,101,102,103,104,105,106,107,108,109,110,111,112,113,114,115,116,117,118,119,120,121,122,123,124,125,126,127,128,129,130,131,132,133,134,135,136,137] are cited in the Supplementary Materials.
