# Supplementary material for: TNFRSF1B Gene Variants in Clinicopathological Aspects and Prognosis of Patients with Cutaneous Melanoma
Source: Int J Mol Sci. 2024 Mar 1;25(5):2868. doi: 10.3390/ijms25052868 (PMC10932403; doi:10.3390/ijms25052868)
Supplement: Supplementary file 1 [file ijms-25-02868-s001.zip › ijms-2846149-Supplementary Material.pdf]

Article – Supplementary Material

TNFRSF1B gene variants in clinicopathological aspects and prognosis of patients with cutaneous melanoma

Bruna Fernandes Carvalho [1], Gabriela Vilas Bôas Gomez [1], Juliana Carron [1], Ligia Traldi Macedo [1,2], Gisele Melo Gonçalves [3], Vinicius de Lima Vazquez [3], Sergio Vicente Serrano [4], Gustavo Jacob Lourenço [1], Carmen Silvia Passos Lima [1,2]

Short title: *TNFRSF1B* gene variants in cutaneous melanoma

[1] Laboratory of Cancer Genetics, School of Medical Sciences, University of Campinas, Campinas, São Paulo, Brazil

[2] Department of Anesthesiology, Oncology, and Radiology, School of Medical Sciences, University of Campinas, Campinas, São Paulo, Brazil

[3] Melanoma and Sarcoma Surgery Department, Barretos Cancer Hospital, Barretos, São Paulo, Brazil

[4] Department of Medical Oncology, Barretos Cancer Hospital, Barretos, São Paulo, Brazil

Corresponding author

Carmen Silvia Passos Lima, MD, PhD

Clinical Oncology Service, Department of Anesthesiology, Oncology and Radiology

School of Medical Sciences, University of Campinas

Rua Alexander Fleming, 181, Cidade Universitária Zeferino Vaz

Barão Geraldo, Campinas, São Paulo, Brazil

CEP: 13083-970

Phone: +55 19 3521-9120

E-mail: carmenl@fcm.unicamp.br

Supplementary table S1. Frequencies of genotypes of the *TNFRSF1B* single nucleotide variants in 433 cutaneous melanoma patients stratified by pathological aspects of the tumor.

Genotypes	Tumor location*		Clinical stage*		Histological type*		Breslow*		Clark level*	
	Head or limbs N (%)	Trunk N (%)	0-II N (%)	III+IV N (%)	Superficial N (%)	Others N (%)	T1+T2 N (%)	T3+T4 N (%)	I-II N (%)	III-V N (%)
**c.587T>G**										
TT	139 (63.5)	123 (63.4)	228 (63.5)	44 (65.7)	162 (63.8)	83 (60.6)	164 (63.3)	100 (63.7)	69 (63.3)	195 (62.9)
TG or GG	80 (36.5)	71 (36.6)	131 (36.5)	23 (34.3)	92 (36.2)	54 (39.4)	95 (36.7)	57 (36.3)	40 (36.7)	115 (37.1)
p value	0.98		0.73		0.53		1.00		0.94	
TT or TG	212 (96.8)	181 (93.3)	342 (95.3)	64 (95.5)	241 (94.9)	132 (96.4)	244 (94.2)	152 (96.8)	103 (94.5)	296 (95.5)
GG	7 (3.2)	13 (6.7)	17 (4.7)	3 (4.5)	13 (5.1)	5 (3.6)	15 (5.8)	5 (3.2)	6 (5.5)	14 (4.5)
p value	0.07		1.00		0.61		0.34		0.79	
**c.*188G>A**										
GG	61 (28.0)	71 (36.6)	118 (33.0)	22 (32.8)	89 (35.0)	41 (30.1)	86 (33.2)	52 (33.3)	36 (33.0)	101 (32.7)
GA or AA	157 (72.0)	123 (63.4)	240 (67.0)	45 (67.2)	165 (65.0)	95 (69.9)	173 (66.8)	104 (66.7)	73 (67.0)	208 (67.3)
p value	0.06		0.98		0.32		1.00		0.94	
GG or GA	171 (78.4)	156 (80.4)	287 (80.2)	52 (77.6)	209 (82.3)	107 (78.7)	204 (78.8)	129 (82.7)	86 (78.9)	249 (80.6)
AA	47 (21.6)	38 (19.6)	71 (19.8)	15 (22.4)	45 (17.7)	29 (21.3)	55 (21.2)	27 (17.3)	23 (21.1)	60 (19.4)
p value	0.62		0.63		0.38		0.37		0.70	
**c.*215T>C**										
TT	86 (39.3)	80 (41.2)	146 (40.7)	28 (41.8)	116 (45.7)	50 (36.5)	107 (41.3)	66 (42.0)	44 (40.4)	128 (41.3)
TC or CC	133 (60.7)	114 (58.8)	213 (59.3)	39 (58.2)	138 (54.3)	87 (63.5)	152 (58.7)	91 (58.0)	65 (59.6)	182 (58.7)

p value	0.68		0.86		0.08		0.91		0.86	
TT or TC	189 (86.3)	176 (90.7)	318 (88.6)	60 (89.6)	228 (89.8)	122 (89.1)	229 (88.4)	143 (91.1)	96 (88.1)	277 (89.4)
CC	30 (13.7)	18 (9.3)	41 (11.4)	7 (10.4)	26 (10.2)	15 (10.9)	30 (11.6)	14 (8.9)	13 (11.9)	33 (10.6)
p value	0.16		0.81		0.82		0.41		0.71	
**c.*922C>T**										
CC	87 (39.7)	70 (36.3)	142 (39.7)	21 (31.3)	89 (35.2)	56 (40.9)	93 (36.0)	62 (39.5)	40 (36.7)	120 (38.8)
CT or TT	132 (60.3)	123 (63.7)	216 (60.3)	46 (68.7)	164 (64.8)	81 (59.1)	165 (64.0)	95 (60.5)	69 (63.3)	189 (61.2)
p value	0.47		0.19		0.26		0.53		0.69	
CC or CT	195 (89.0)	166 (86.0)	316 (88.3)	58 (86.6)	221 (87.4)	122 (89.1)	221 (85.7)	142 (90.4)	94 (86.2)	272 (88.0)
TT	24 (11.0)	27 (14.0)	42 (11.7)	9 (13.4)	32 (12.6)	15 (10.9)	37 (14.3)	15 (9.6)	15 (13.8)	37 (12.0)
p value	0.35		0.68		0.62		0.17		0.62	

N: number of patients; %: percentage. Breslow thickness: T1: ≤ 1.00 mm, T2: > 1.00-2.00 mm, T3: > 2.01-4.00 mm, and T4: > 4.00 mm. [*]The number of individuals differs from the initial one as some data were not included in their medical records. Nomenclatures of c.*188G>A (known as c.*188A>G) and c.*215T>C (known as c.*215C>T) singe nucleotide variants were modified in the present study due to the higher frequencies of G and C alleles in our population, respectively.

Supplementary table S2. Nonsignificant genotypes and haplotypes of the *TNFRSF1B* single nucleotide variants in survival of 255 cutaneous melanoma patients.

Variable	Univariate Cox regression						Multivariate Cox regression			
	N total/ N event	PFS HR (95% CI)	*p* value	N total/ N event	MSS HR (95% CI)	*p* value	PFS adjusted HR (95% CI)	*p* value	MSS adjusted HR (95% CI)	*p* value
**c.587T>G**										
TT	161/53	Reference	0.53	161/38	Reference	0.68	NA		NA	
TG or GG	94/34	1.14 (0.74-1.76)		94/23	1.11 (0.66-1.87)					
TT or TG	243/82	Reference	0.48	243/57	Reference	0.36	NA		NA	
GG	12/5	1.37 (0.55-3.40)		12/4	1.60 (0.58-4.43)					
**c.*188G>A**										
GG	85/24	Reference	0.11	85/14	Reference	0.05	Reference	0.55	Reference	0.23
GA or AA	170/63	1.45 (0.91-2.33)		170/47	1.83 (1.00-3.33)		1.18 (0.67-2.06)		1.52 (0.76-3.06)	
GG or GA	201/70	Reference	0.67	201/48	Reference	0.92	NA		NA	
AA	54/17	1.12 (0.52-1.51)		54/13	1.03 (0.55-1.90)					
**c.*215T>C**										
TT	103/34	Reference	0.92	103/23	Reference	0.90	NA		NA	
TC or CC	152/53	1.02 (0.63-1.50)		152/38	1.03 (0.61-1.73)					
TT or TC	226/78	1.16 (0.43-1.71)	0.66	226/54	1.08 (0.42-2.03)	0.84	NA		NA	
CC	29/9	Reference		29/7	Reference					
**c.587T>G + c.*188G>A**										
TT + GG	48/11	Reference	0.07	48/5	Reference	0.04	Reference	0.56	Reference	0.23
TG or GG + GA or AA	57/21	1.94 (0.93-4.04)		57/14	2.86 (1.02-7.96)		1.28 (0.54-3.07)		2.03 (0.62-6.63)	

TT or TG + GG or GA	191/65	Reference	NC	191/44	Reference	NC	NA		NA	
GG + AA	2/0	NC		2/0	NC					
**c.587T>G + c.*215T>C**										
TT + TT	59/15	Reference	0.65	59/11	Reference	0.67	NA		NA	
TG or GG + TC or CC	50/15	1.17 (0.57-2.40)		50/11	1.19 (0.51-2.75)					
TT or TG + TT or TC	216/73	Reference	NC	216/50	Reference	NC	NA		NA	
GG + CC	2/0	NC		2/0	NC					
**c.*188G>A + c.*215T>C**										
GG + TT	50/15	Reference	0.38	50/9	Reference	0.23	NA		NA	
GA or AA + TC or CC	117/44	1.30 (0.72-2.33)		117/33	1.56 (0.74-3.28)					
GG or GA + TT or TC	188/68	1.17 (0.53-2.55)	0.68	188/47	1.39 (0.59-3.25)	0.44	NA		NA	
AA + CC	16/7	Reference		16/6	Reference					
**c.*188G>A + c.*922C>T**										
GG + CC	34/9	Reference	0.12	34/6	Reference	0.10	Reference	0.18	Reference	0.08
GA or AA + CT or TT	105/43	1.75 (0.85-3.60)		105/34	2.05 (0.86-4.88)		1.73 (0.76-3.95)		2.33 (0.88-6.17)	
GG or GA + CC or CT	183/61	4.31 (0.03-1.67)	0.14	183/41	Reference	NC	NC	0.99	NA	
AA + TT	11/1	Reference		11/0	NC		Reference			
**Haplotype**										
TGTC	165/56	1.04 (0.67-1.61)	0.85	165/38	1.17 (0.70-1.98)	0.53	NA		NA	
Others	89/31	Reference		89/23	Reference					
TACC	103/39	1.16 (0.56-1.31)	0.49	103/29	1.26 (0.47-1.31)	0.36	NA		NA	
Others	151/48	Reference		151/32	Reference					

N: number of patients; PFS: progression-free survival; MSS: melanoma-specific survival; HR: relative risks of events; CI: confidence interval; NA: characteristics not included in multivariate analysis; NC: not calculated. Nomenclatures of c.*188G>A (also known as c.*188A>G) and c.*215T>C (also known as c.*215C>T) single nucleotide variants (SNVs) change due to the higher frequency of G and T alleles in our population, respectively. Haplotypes represent alleles of the *TNFRSF1B* c.587T>G, c.*188G>A, c.*215T>C, and c.*922C>T SNVs, respectively. Significant clinical aspects in the univariate Cox analysis were included in the multivariate analysis.

Supplementary table S3. Thirty-eight single nucleotide variants located in the *TNFRSF1B* gene identified through studies published in the PubMed Central database.

SNV	Gene location	Previous study	Reference
**rs652625**	Promoter	Affect levels of receptors I and II, lung cancer, and diabetes mellitus	[29,77]
rs5745946	Promoter	Migraine	[78]
rs652625	Promoter	Rheumatoid arthritis	[79]
**rs1061622**	CDS	Schizophrenia, liver diseases, autoimmune disease, cervical cancer, rheumatoid arthritis, Crohn's disease, lung cancer, pre-eclampsia, cardiac insufficiency, non-Hodgkin's lymphoma, chagas disease, non-melanoma skin, interleukin-1 receptor 2 interaction with toll-type receptor 4, tuberculosis, epidermoid esophageal carcinoma, and diabetes mellitus	[29,80-93]
**rs5746026**	CDS	Cervical cancer, and lung cancer	[86,91]
**rs17883432**	CDS	Cervical cancer, and diabetes mellitus	[91,94]
rs616645	Intron	Depression	[95]
**rs945439**	Intron	Cervical cancer, and diabetes mellitus	[91]
rs472093	Intron	Cervical cancer	[91]
rs976881	Intron	Crohn's disease	[96]
rs496888	Intron	Diabetes mellitus	[94]
rs6697733	Intron	Diabetes mellitus	[94]
rs235249	Intron	Diabetes mellitus	[94]
rs17884213	Intron	Diabetes mellitus	[94]
rs3766730	Intron	Interleukin-1 receptor 2 interaction with toll-type receptor 4	[27]
rs616645	Intron	Interleukin-1 receptor 2 interaction with toll-type receptor 4	[27]

rs816050	Intron	Interleukin-1 receptor 2 interaction with toll-type receptor 4	[27]
rs474247	Intron	Interleukin-1 receptor 2 interaction with toll-type receptor 4	[27]
rs1201157	Intron	Interleukin-1 receptor 2 interaction with toll-type receptor 4	[27]
rs5746051	Intron	Interleukin-1 receptor 2 interaction with toll-type receptor 4	[27]
rs5746053	Intron	Interleukin-1 receptor 2 interaction with toll-type receptor 4	[27]
rs235219	Intron	Interleukin-1 receptor 2 interaction with toll-type receptor 4	[27]
rs597519	Intron	Rheumatoid arthritis	[79]
rs976881	Intron	Osteoporosis	[97]
rs5746059	Intron	Obesity	[98]
**rs945439**	**Intron**	Bone structural defects	[99]
**rs3397**	3'-URT	Schizophrenia, cervical cancer, Crohn's disease, tuberculosis, epidermoid esophageal carcinoma, and diabetes mellitus	[31,80,81,88,91,93,100]
**rs1061624**	3'-UTR	Schizophrenia, liver diseases, colorectal cancer, Crohn's disease, lung cancer, interleukin-1 receptor 2 interaction with toll-type receptor 4, and epidermoid esophageal carcinoma	[27,29,31,81,88,92,93,101]
**rs1061628**	**3'-UTR**	T cell lymphoma	[27]
rs1061631	3'-UTR	Interleukin-1 receptor 2 interaction with toll-type receptor 4	[27]
rs5030792	3'-UTR	Premature birth	[102]
rs42686850	NA	TNFR2 gene expression in cows	[103]
rs590368	NA	Gene polymorphisms affect levels of receptors I and II	[77]
rs136228480	NA	TNFR2 gene expression in cows	[104]
rs522807	NA	Interleukin-1 receptor 2 interaction with toll-type receptor 4	[27]

rs235214	NA	Interleukin-1 receptor 2 interaction with toll-type receptor 4	[27]
rs72863489	NA	Premature birth	[102]
rs520916	NA	Rheumatoid arthritis	[79]

SNV: single nucleotide variant; rs: reference number of SNV; NA: not analyzed due to the lack of information in the database; CDS: coding sequence; UTR: untranslated region. Data was obtained from dbSNP database (http://www.ncbi.nlm.nih.gov/projects/SNP). The nine selected SNVs are presented in bold letters.

Supplementary table S4. Association of *TNFRSF1B* single nucleotide variants with microRNA binding sites and their consequences in tumor cells.

SNV	microRNA	Seed-match site	Consequence on tumor [reference]
c.*188A>G	miR-99a 5p	5mer	Acts as tumor suppressor in urothelial carcinoma and oral carcinoma [105,106]
	miR-100-5p	5mer	It confers resistance to cisplatin in lung cancer [107]
	miR-549	Not rated	No studies on the subject were found in the literature
	**miR-639**	7mer-m8	Promotes thyroid and breast cancer proliferation [108,109]
	miR-720	Not rated	No studies on the subject were found in the literature
	miR-922	Not rated	No studies on the subject were found in the literature
	miR-3126-5p	Not rated	No studies on the subject were found in the literature
	miR-4771	Not rated	No studies on the subject were found in the literature
c.*215C>T	miR-122-3p	7mer-m8	Promotes the development of hepatocellular carcinoma [110]
	miR-220	Not rated	No studies on the subject were found in the literature
	**miR-329-3p**	7mer-m8	Associated with worse cervical cancer prognosis [111]
	miR-329 5p	Not rated	No studies on the subject were found in the literature
	**miR-362-3p**	7mer-m8	Inhibits proliferation of hepatocellular carcinoma [112]
	miR-423-5p	5mer	Inhibits ovarian cancer and osteosarcoma [113-115]
	miR-603	6mer	Inhibits breast cancer [116] and promotes glioma growth [117]
	miR-767-5p	Not rated	No studies on the subject were found in the literature
	miR-1228-3p	6mer	Promotes breast cancer growth [118]
	miR-1302	5mer	Related to prostate cancer [119]
	miR-3126-5p	Not rated	No studies on the subject were found in the literature
	miR-4523	Not rated	No studies on the subject were found in the literature
c.*922C>T	**miR-96-5p (miR-96)**	6mer	Promotes hepatocellular carcinoma invasion and glioma growth [120-122], regulates apoptosis in bladder cancer [123], and radioresistance in esophageal cancer [124]
	miR-182-5p	6mer	Inhibits retinoblastoma invasion [125] and it confers radioresistance in lung cancer [126]

miR-374b-3p	6mer	Promotes gastric cancer metastases [127] and inhibits the proliferation of colon cancer [128]
miR-382	Not rated	No studies on the subject were found in the literature
miR-382-5p	5mer	Aggravates the progression of breast cancer [129]
miR-524-5p	Not rated	No studies on the subject were found in the literature
miR-571	Not rated	No studies on the subject were found in the literature
miR-548	Not rated	No studies on the subject were found in the literature
miR-1179	6mer	Inhibits glioblastoma [130] and lung cancer [131] proliferation
miR-1256	6mer	Inhibits the proliferation of lung cancer [132]
miR-1258	5mer	Inhibits proliferation of hepatocellular carcinoma [133] and suppresses the progression of lung cancer, ovarian cancer and gastric cancer [108,134,135]
**miR-1271-5p (miR-1271)**	6mer	Inhibits invasion by pancreatic, lung, prostate, and colorectal cancer [55-57,136]
miR-1306-3p	6mer	Promotes metastasis of hepatocellular carcinoma [137]
miR-4715-5p	Not rated	No studies on the subject were found in the literature

SNV: single nucleotide variant; miR: microRNA. Selected miRNAs are shown in bold letters.

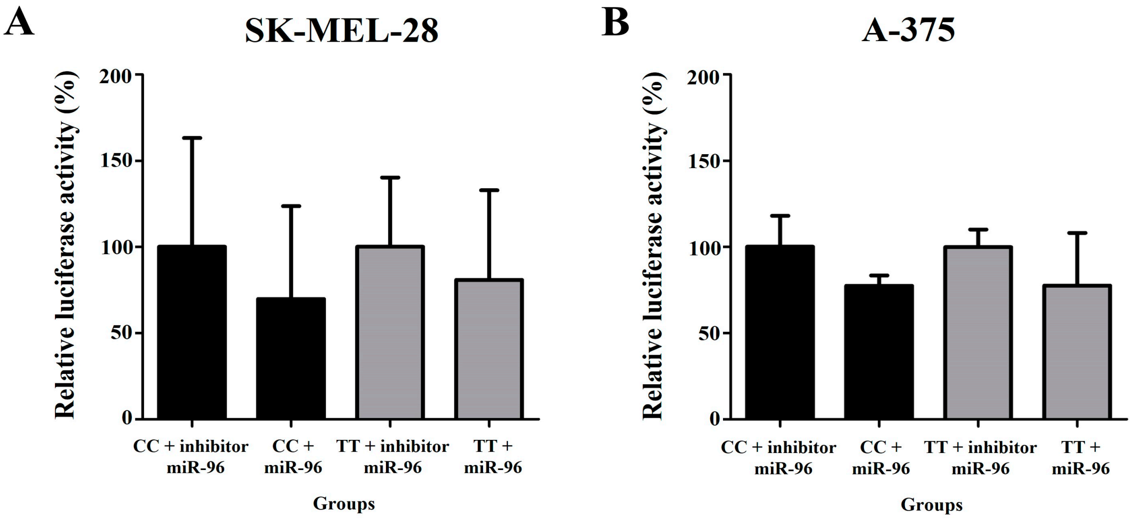

Supplementary figure S1. Quantification of the relative luciferase activity in the groups: pMIR c.*922C (CC genotype) co-transfected with the microRNA inhibitor (miR)-96 (considered 100% luciferase enzyme activity); pMIR c.*922C co-transfected with the mimic sequence of miR-96; pMIR c.*922T (TT genotype) co-transfected with the inhibitor of miR-96; and pMIR c.*922T group co-transfected with the mimic sequence of miR-96 in melanoma cell lines SK-MEL-28 (**A**) and A-375 (**B**). The relative activity of luciferase did not show significant differences when comparing the group containing the plasmid with the CC genotype co-transfected with the inhibitory sequence of miR-96 and the group containing the same plasmid co-transfected with the mimic sequence of miR-96 (SK-MEL-28: 100.0 *versus* 70.0%, $p = 0.23$; A-375: 100.0 *versus* 79.0%, $p = 0.05$). Also, no significant differences were observed when comparing the group containing the plasmid with the CC genotype co-transfected with the miR-96 mimic sequence and the group containing the plasmid with the TT genotype co-transfected with the miR-96 mimic sequence (SK-MEL-28: 70.0 *versus* 81.0%, $p = 0.49$; A-375: 79.0 *versus* 78.0%, $p = 0.25$).

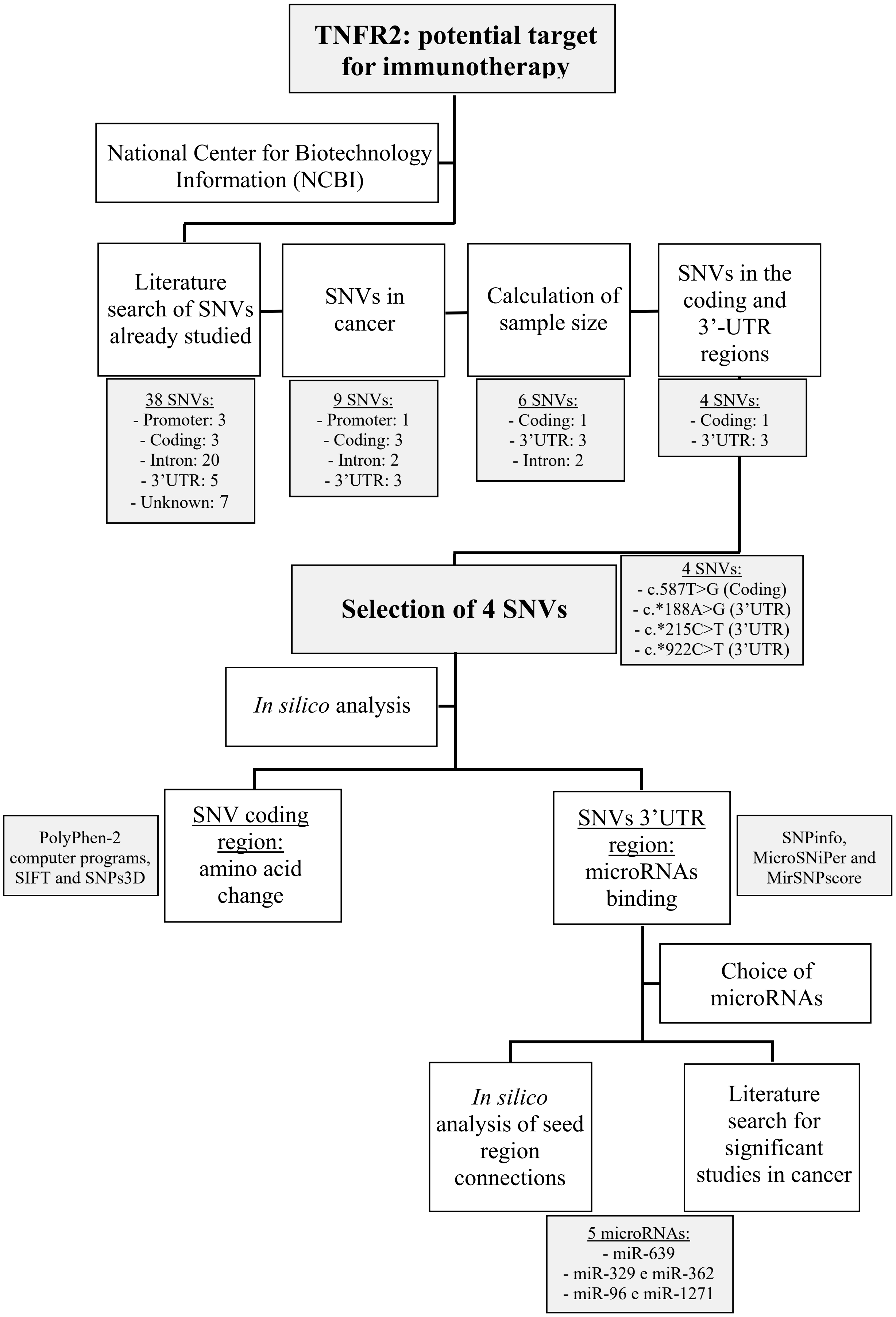
TNFR2: potential target for immunotherapy
National Center for Biotechnology Information (NCBI)
Literature search of SNVs already studied
38 SNVs:
- Promoter: 3
- Coding: 3
- Intron: 20
- 3'UTR: 5
- Unknown: 7
SNVs in cancer
9 SNVs:
- Promoter: 1
- Coding: 3
- Intron: 2
- 3'UTR: 3
Calculation of sample size
6 SNVs:
- Coding: 1
- 3'UTR: 3
- Intron: 2
SNVs in the coding and 3'-UTR regions
4 SNVs:
- Coding: 1
- 3'UTR: 3
Selection of 4 SNVs
4 SNVs:
- c.587T>G (Coding)
- c.*188A>G (3'UTR)
- c.*215C>T (3'UTR)
- c.*922C>T (3'UTR)
In silico analysis
PolyPhen-2 computer programs, SIFT and SNPs3D
SNV coding region: amino acid change
SNVs 3'UTR region: microRNAs binding
SNPinfo, MicroSNiPer and MirSNPscore
Choice of microRNAs
In silico analysis of seed region connections
Literature search for significant studies in cancer
5 microRNAs:
- miR-639
- miR-329 e miR-362
- miR-96 e miR-1271

Supplementary figure S2. Flowchart of the process used to select single nucleotide variants (SNVs) for the study. The National Center for Biotechonology Information database was searched to find SNVs of interest in the *TNFRSF1B* gene and 38 SNVs were identified. Next, nine SNVs previously associated with tumors were selected from the 38 SNVs, and six SNVs with sample sizes compatible with the number of samples available at the Cancer Genetics Laboratory of University of Campinas (N = 550) remained in the study. Subsequently, one SNV located in the amino acid coding region and three SNVs located in in the 3'-UTR region of the *TNFRSF1B* gene were selected for the study due to potential structural alteration of the receptor and possible alteration of binding sites of miRNA and modulation of gene expression, respectively. Finally, miRNAs associated with SNVs and with possible consequences in tumor cells were selected from the literature.

A

***TNFRSF1B* c.*188A>G**

CUGCAGGCCAAGAGCAGAGGCAGCG**A**GUUGUGGAAAGCCUCUGCUGCCAUG

| | | | | | |

UGUCGCGAGCGUUGGCGUCGCUA – miR-639 (7mer-m8)

***TNFRSF1B* c.*188A>G**

CUGCAGGCCAAGAGCAGAGGCAGCG**G**GUUGUGGAAAGCCUCUGCUGCCAUG

| | | | | |

UGUCGCGAGCGUUGGCGUCGCUA – miR-639

B

***TNFRSF1B* c.*215C>T**

UUGUGGAAAGCCUCUGCUGCCAUGG**C**GUGUCCCUCUCGGAAGGCUGGCUGG

| | | | | |

UUUCUCCAAUUGGUCCACACAA – miR-329-3p

***TNFRSF1B* c.*215C>T**

UUGUGGAAAGCCUCUGCUGCCAUGG**U**GUGUCCCUCUCGGAAGGCUGGCUGG

| | | | | | |

UUUCUCCAAUUGGUCCACACAA – miR-329-3p (7mer-m8)

C

***TNFRSF1B* c.*215C>T**

UUGUGGAAAGCCUCUGCUGCCAUGG**C**GUGUCCCUCUCGGAAGGCUGGCUGG

| | | | | |

ACUUAGGAACUUAUCCACACAA – miR-362-3p

***TNFRSF1B* c.*215C>T**

UUGUGGAAAGCCUCUGCUGCCAUGG**U**GUGUCCCUCUCGGAAGGCUGGCUGG

| | | | | | |

ACUUAGGAACUUAUCCACACAA – miR-362-3p (7mer-m8)

D

***TNFRSF1B* c.*922C>T**

AAAAAAAAGCACCGCCUCCAAAUGC**C**AACUUGUCCUUUUGUACCAUGGUGU

| | | | | |

UCGUUUUUACACGAUCACGGUUU – miR-96 (6mer)

***TNFRSF1B* c.*922C>T**

AAAAAAAAGCACCGCCUCCAAAUGC**U**AACUUGUCCUUUUGUACCAUGGUGU

| | | | |

UCGUUUUUACACGAUCACGGUUU – miR-96

E

***TNFRSF1B* c.*922C>T**

AAAAAAAAGCACCGCCUCCAAAUGC**C**AACUUGUCCUUUUGUACCAUGGUGU

| | | | | |

ACUCACGAACGAUCCACGGUUC – miR-1271 (6mer)

***TNFRSF1B* c.*922C>T**

AAAAAAAAGCACCGCCUCCAAAUGC**U**AACUUGUCCUUUUGUACCAUGGUGU

| | | | |

ACUCACGAACGAUCCACGGUUC – miR-1271

Supplementary figure S3. MicroRNA binding sites (miRNAs) located in the 3'-untranslated (3'UTR) region of the *TNFRSF1B* gene related to c.*188A>G, c.*215C>T and c.* 922C>T (in bold) single nucleotide variants (SNVs). The underlined part refers to the "seed" binding region of the miRNA. (**A**) miR-639 has a 7mer-m8 binding site with the 3'-UTR region of the A allele of c.*188A>G SNV. (**B**) The microRNA miR-329-3p has a binding site of 7 nucleotides and pairing with the target at position 8 (7mer-m8) with the 3'-UTR region of the T allele of c.*215C>T SNV. (**C**) miR-362-3p has a 7mer-m8 binding site with the 3'-UTR region of the T allele of c.*215C>T SNV. (**D**) miR-96 has a binding site of 6 nucleotides (6mer) with the 3'-UTR region of the C allele of c.*922C>T SNV. (**E**) miR-1271 has a 6mer binding site with the 3'-UTR region of the C allele of c.*922C>T SNV.
